# Early fiberoptic bronchoscopy during non-invasive ventilation in patients with decompensated chronic obstructive pulmonary disease due to community-acquired-pneumonia

**DOI:** 10.1186/cc8993

**Published:** 2010-04-29

**Authors:** Raffaele Scala, Mario Naldi, Uberto Maccari

**Affiliations:** 1 UO Pneumologia, Unità di Terapia Semi-Intensiva Respiratoria, Endoscopia Toracica, Ospedale S. Donato, Via P. Nenni 20 52100, Arezzo, Italy

## Abstract

**Introduction:**

Inefficient clearance of copious respiratory secretion is a cause of non-invasive positive pressure ventilation (NPPV) failure, especially in chronic respiratory patients with community-acquired-pneumonia (CAP) and impaired consciousness. We postulated that in such a clinical scenario, when intubation and conventional mechanical ventilation (CMV) are strongly recommended, the suction of secretions with fiberoptic bronchoscopy (FBO) may increase the chance of NPPV success. The objective of this pilot study was, firstly, to verify the safety and effectiveness of early FBO during NPPV and, secondly, to compare the hospital outcomes of this strategy versus a CMV-based strategy in patients with decompensated chronic obstructive pulmonary disease (COPD) due to CAP who are not appropriate candidates for NPPV because of inefficient mucous clearance and hypercapnic encephalopathy (HE).

**Methods:**

This is a 12-month prospective matched case-control study performed in one respiratory semi-intensive care unit (RSICU) with expertise in NPPV and in one intensive care unit (ICU). Fifteen acutely decompensated COPD patients with copious secretion retention and HE due to CAP undergoing NPPV in RSICU, and 15 controls (matched for arterial blood gases, acute physiology and chronic health evaluation score III, Kelly-Matthay scale, pneumonia extension and severity) receiving CMV in the ICU were studied.

**Results:**

Two hours of NPPV significantly improved arterial blood gases, Kelly and cough efficiency scores without FBO-related complications. NPPV avoided intubation in 12/15 patients (80%). Improvement in arterial blood gases was similar in the two groups, except for a greater PaO2/fraction of inspired oxygen ratio with CMV. The rates of overall and septic complications, and of tracheostomy were lower in the NPPV group (20%, 20%, and 0%) versus the CMV group (80%, 60%, and 40%; *P *< 0.05). Hospital mortality, duration of hospitalisation and duration of ventilation were similar in the two groups.

**Conclusions:**

In patients with decompensated COPD due to CAP who are candidates for CMV because of HE and inability to clear copious secretions, NPPV with early therapeutic FBO performed by an experienced team is a feasible, safe and effective alternative strategy.

## Introduction

Non-invasive positive pressure ventilation (NPPV) is the first-line treatment of hypercapnic acute respiratory failure (ARF) in severe chronic obstructive pulmonary disease (COPD) exacerbations. Compared with standard medical therapy, it reduces the rate of endotracheal intubation (ETI) and the associated complications, as well as the mortality and length of stay in hospital [1,2]. However, the inefficacy to spontaneously clear airways from an excessive burden of respiratory secretions is likely to cause NPPV failure [3,4]. This is due to the kinds of interfaces used to deliver NPPV, which do not allow direct access into the airways. Conversely, during conventional mechanical ventilation (CMV) respiratory secretions may be easily aspirated via the endotracheal tube.

This is the reason why most randomised controls trials (RCTs) of NPPV for ARF have excluded patients without an efficient clearance of secretions. As a matter of fact, the inability to spontaneously remove respiratory secretions has been considered a relative contraindication to start NPPV in ARF, especially if it occurs in patients with impaired consciousness and depressed cough [1,2]. In fact, the coexistence of abundant secretions and sensorium depression 'triggers' a vicious circle that leads to a progressive clinical-physiological deterioration, which eventually requires ETI.

Few published data suggested that some non-invasive physiotherapeutic techniques may improve mucous clearance in COPD exacerbations managed with NPPV [5-7]. However, to our knowledge, no specific studies have addressed the feasibility and usefulness of any available mucous clearance strategies in patients who are not eligible for NPPV because of their incapability to spontaneously eliminate accumulated secretions associated with hypercapnic encephalopathy (HE).

Recent studies have shown that, within expert units, NPPV is feasible and may be applied with success in moderate-to-severe HE due to COPD exacerbations with a similar short- and long-term survival but fewer septic complications compared with CMV [8-10]. Moreover, despite the uncertain role of NPPV to successfully treat severe community-acquired pneumonia (CAP) in *de novo *hypoxaemic ARF patients [1,2], one RCT demonstrated the effectiveness of NPPV in reducing the rate of ETI in hypercapnic COPD exacerbations precipitated by CAP [11]. Furthermore, accumulated evidence supports the feasibility and safety of performing a diagnostic fiberoptic bronchoscopy (FBO) with broncho-alveolar lavage (BAL) for a suspected pneumonia under the assistance of NPPV in patients with either pure hypoxaemic or hypercapnic ARF [12-17].

We postulated that in the clinical scenario of patients with COPD decompensations secondary to CAP who require ETI and ventilatory assistance because of impaired mucous clearance and HE, the early suction of secretions with FBO performed during NPPV by an expert team is feasible. It may also allow for the successful expanded application of NPPV. The choice of including episodes of CAP in place of simple exacerbations of COPD was based on the intention of 'stressing' the model of bronchial hypersecretion to test the feasibility of this new FBO-NPPV approach.

We therefore performed this pilot study with the aims, firstly, to verify the safety and effectiveness of early therapeutic FBO to clear airways during NPPV and, secondly, to compare the hospital outcomes of this strategy administered in a respiratory semi-intensive care unit (RSICU) with the CMV-based strategy provided in the ICU to treat ARF episodes occurring in COPD patients who were not appropriate candidates for NPPV because of their inability to remove copious secretion and HE due to CAP-triggered decompensations.

## Materials and methods

### Study design

This prospective case-control study was performed between January and December 2008 in two centres: a three-bed RSICU in the Respiratory Division and the eight-bed general ICU of S. Donato Hospital, Arezzo, Italy. The study protocol was approved by the ethics committee and by the institutional review board and was performed in accordance with the ethical standards laid down in the 1964 Declaration of Helsinki. Due to the depressed mental status of the patients, the informed written consent was obtained from their next of kin. The decision-making attending physicians of both centres were not aware of their participation in the study because the strategy was based on early FBO during both NPPV and CMV in ARF patients with impaired mucous clearance, which had been part of their clinical practice for several years.

### Cases (NPPV group)

All consecutive COPD patients [18] with ARF precipitated by severe CAP [19], showing depressed sensorium and bronchial hypersecretion, admitted to the RSICU over the observed period were considered eligible for the study. Diagnosis of CAP was radiographically confirmed in all cases while its severity was ascertained according to the presence of at least three criteria recommended by the Infective Diseases Society of America (IDSA) and the American Thoracic Society (ATS) guidelines [19]. To be included in the study, the patients had to meet all the following criteria while breathing oxygen via a venturi mask: (a) pH less than 7.33 and partial pressure of arterial carbon dioxide (PaCO_2_) above 55 mmHg; (b) partial pressure of arterial oxygen (PaO_2_)/fraction of inspired oxygen (FiO_2_) ratio less than 250; (c) dyspnoea at rest with respiratory rate (RR) above 25 breaths/min; (d) use of accessory respiratory muscles or paradoxical abdominal breathing; (e) HE as assessed by the Kelly-Matthay score between two and four [20]; (f) inability to spontaneously clear airways from excessive secretions, as expressed by the lowest score of an arbitrary cough efficiency scale evaluated by the nurses on the basis of the volume of the expelled sputum after three hard coughing efforts (1 = less than 2 ml; 2 = between 2 and 6 ml and; 3 = more than 6 ml) [21]. Due to the poor patient's cooperation, more objective tools for the assessment of cough strength (i.e. peak expiratory cough flow) were not applied.

It should be noted that before the implementation of the FBO-NPPV protocol in the RSICU clinical practice all the potential eligible cases had been intubated and transferred to the ICU due to the high likelihood of NPPV failure associated with depressed cough and sensorium [1,2].

Exclusion criteria from the FBO-NPPV protocol were: (a) refusal of NPPV; (b) facial deformity sufficient to preclude mask fitting; (c) preexisting psychiatric and/or neurological disorders unrelated to HE; (d) upper gastrointestinal bleeding; (e) upper airway obstruction; (f) acute coronary syndromes; (g) tracheostomy or ETI before admission; (h) need for urgent ETI due to cardiac or respiratory arrest or psychomotor agitation, severe haemodynamic instability [10]. The two major criteria recently suggested by IDSA/ATS guidelines [19] for the ICU admission of patients with severe CAP (i.e. mandatory CMV due to the reasons detailed in point h of the guidelines and septic shock with need for vasopressors) were also considered reasons of exclusion from the FBO-NPPV protocol.

#### Before FBO

NPPV (Vela, Viasys, Loma Linda, CA, USA) was delivered in pressure support (PS) mode with positive end-expiratory pressure (PEEP) via a well-fitting full-face mask (Mirage, ResMed, San Diego, CA, USA) with the addition of a heated humidifier (DAR HC 2000, Mallinckrodt DAR, Mirandola, Italy). PS was initially set at 10 cmH_2_O and then titrated to achieve an expiratory tidal volume of 8 to 10 ml/kg and a RR below 25 breaths/min to a maximum of 25 cmH_2_O depending on clinical and arterial blood gases (ABGs) response and patient tolerance. PEEP was always set at 5 cmH_2_O [10]. Back-up RR was set at a 14 to 18 breaths/min, lower than the patient's spontaneous RR. FiO_2 _was initially set at 0.70.

#### During FBO

**Figure 1:**
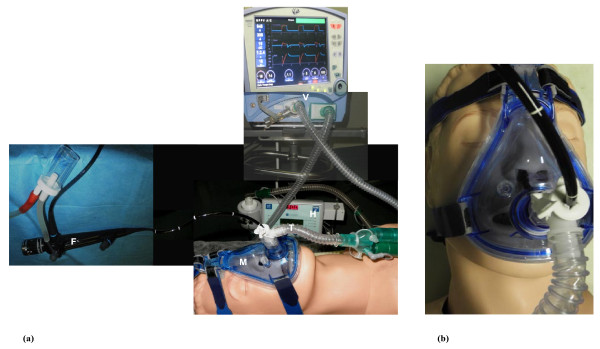
**Fiberoptic bronchoscopy**. **(a) **The fiberoptic bronchoscopy (FBO)-noninvasive positive pressure ventilation (NPPV) procedure. **(b) **The trans-nasally introduction of the bronchoscope. F = fiberoptic bronchoscope; H = heated humidifier; M = mask; T = T-adapter; V = ventilator.

FBO was performed after the patients had adapted to NPPV. A T-adapter was attached to the face mask for the insertion of FBO (model P40; Olympus; Tokyo, Japan) via a nasal route [14] (Figure 1). During the bronchoscopic procedure, the FiO_2 _was kept at 1.0. Topical anaesthesia of the nasopharynx (10% lidocaine spray solution) and larynx (2% lidocaine hydrochloride, not exceeding an overall does of 200 mg) was performed before advancing the bronchoscope into the tracheobronchial tree [14]. No pharmacological sedation and/or analgesia was administered during the FBO-NPPV procedure. Firstly, a careful suction of bronchial secretions was performed to fully clear airways. Then, the tip of the FBO was wedged into the bronchial subsegment, which was tributary of the lung consolidation showed by the chest radiograph. BAL was performed by sequential instillation of five aliquots of 30 mL non-bacteriostatic saline solution at room temperature. The retrieved fluid was sent immediately to the microbiology laboratory for microscopic analysis and culturing. The isolated bacteria with a count of 10^4 ^cfu/mL or more of the BAL fluid were considered as the aetiological agents of CAP [22].

#### After FBO

After the bronchoscopic procedures, the FiO_2 _was decreased in order to maintain the pulse oximetry (SpO_2_) at 90 to 94% and PS levels were adjusted according to the criteria described in the pre-FBO phase.

NPPV was provided by nurses and physicians adequately trained with 12 years of accumulated experience with this technique in the RSICU following yearly courses aiming to educate naïve units, update on newly introduced equipment, and share internal protocols. The mean nurse:patient ratio in the overall respiratory division was 1:6, with one nurse for each shift dedicated to the RSICU [10]. Routine chest physiotherapy was available to facilitate expectoration. Electrocardiogram, SpO_2_, and noninvasive blood pressure were monitored continuously. ABGs were sampled as follows: at baseline; before, during, and at the end of FBO (i.e. after two hours of NPPV); after four hours of NPPV; and subsequently as clinically indicated. Prompt ETI with transfer to the ICU was available at any time during NPPV treatment.

NPPV was applied continuously at least during the first 12 to 24 hours. Once clinical status, Kelly-Matthay score, and ABGs improved, NPPV was administered intermittently with sessions lasting two to six hours three times daily [10]. Then PS was reduced progressively twice a day by 3 cmH_2_O until a level of 8 cmH_2_O or less was reached. NPPV weaning was considered successful after three days of ventilation or more when all the following criteria were met for longer than 24 hours while on breathing with oxygen (FiO_2 _0.28): pH above 7.35, SpO_2 _above 90%, RR less than 30 breaths/min, Kelly-Matthay score 1, cough score 3, radiographic improvement of CAP, and stable haemodynamic status [10].

NPPV was considered to have failed if at least one of the following criteria for ETI was met: cardiac arrest or severe haemodynamic instability; respiratory arrest or gasping; mask intolerance; lack of improvement in cough score; and/or worsening of ABGs or of sensorium level during NPPV [10]. Tracheostomy was performed in intubated patients after NPPV failure when the weaning process was prolonged for more than 12 days. Patients who failed with NPPV were promptly intubated in the RSICU but they had to be moved to the general ICU because the low monitoring level and nurse-to-patient ratio available in the respiratory unit could not allow the safely management of invasively ventilated patients.

#### Medical therapy

All patients received standard medical therapy consisting of: controlled oxygen therapy during NPPV-free periods; salbutamol and anticholinergic drugs during NPPV via a spacer; intravenous aminophylline and corticosteroids; subcutaneous low-molecular weight heparin; and therapy for comorbidities if necessary. Antibiotic strategy was based on empirical intravenous administration of levofloxacin plus β-lactam (for penicillin-allergic patients: levofloxacin plus aztreonam), unless some risk factors for *Pseudomonas aeruginosa *were identified (ciprofloxacin plus anti-pseudomonal β-lactam) [19]. Antibiotic-therapy was later eventually adjusted to the results of bacterial cultures and antibiogram according to a de-escalation strategy and/or a drug resistance.

### Controls (CMV group)

Controls were selected from all COPD patients consecutively admitted to the ICU during the same period who received CMV according to the same inclusion criteria used for the NPPV group, and whose data were prospectively collected. Specifically, the nurses who worked in the ICU applied the same assessment scores, cough efficiency included, used in the RSICU. CMV patients showing any of the NPPV group exclusion criteria except for refusal of NPPV or facial deformity sufficient to preclude mask fitting were not included in the study to prevent a potential bias of selection [10]: inclusion in previous studies, major criteria of ICU admission for severe CAP [19] and ETI after the failure of an initial NPPV trial were considered further reasons for exclusion. The matching of controls was performed manually according to the following criteria: PaO_2_/FiO_2 _(± 10), PaCO_2 _(± 5 mmHg), pH (± 0.03), Kelly-Matthay score (± 0), the severity and the extension of CAP according to the CURB-65 (confusion, urea, respiratory rate, blood pressure, age 65 or over) score [19] and the number of lobes radiographically involved (± 0) before mechanical ventilation (MV), respectively; acute physiology and chronic health evaluation (APACHE) score III [23] (± 5 points) assessed within the first 24 hours after admission. When more than one potential control was present, the best matched subject was selected.

The standard therapy protocol was the same as that described for the NPPV group [10] except that controls were sedated at the time of intubation (2 mg/kg propofol intravenously followed by a continuous infusion at 0.5 to 3 mg/kg per hour usually lasting for 24 to 36 hours); no paralysing drugs were used. The ICU nurse:patient ratio was 1:2. CMV was delivered via an ICU ventilator (Siemens 300, Siemens, Berlin, Germany, or Puritan Bennett 840, Covidien, Dublin, Ireland) in assist-control mode (tidal volume 8 to 10 ml/kg; back-up RR 10 to 14 breaths/min; FiO_2 _0.70; PEEP 5 cmH_2_O). In addition to the usual nursing addressed to the suction of secretions through the endotracheal tube and to the routinely applied chest physiotherapy, FBO with BAL was performed in the CMV group within one hour after ETI following the same procedure and adjustment of FiO_2 _used in the NPPV group. When spontaneous breathing reappeared (usually ≥ 24 hours), the ventilator mode was switched to PS [24], following the same criteria used for the NPPV group. Extubation was performed if the patient was able to tolerate a one to two hour T-piece trial with FiO_2 _0.28 (pH > 7.35, SpO_2 _> 90%, RR < 30 breaths/min, normal sensorium, efficient cough, radiographic CAP improvement, stable haemodynamic status). If after 12 days the patient was still intubated and ventilated, tracheostomy was performed according to the judgement of the physician in charge; then weaning was resumed following the above protocol.

### Data collection and end-points

In addition to the matching variables, other parameters were collected: age, gender, body mass index, spirometry and ABGs in a stable status within the previous six months, number of exacerbations experienced in the previous year, comorbidities as assessed by the Charlson score [25], do not resuscitate (DNR) order, antibiotic use in the previous three months, RR, heart rate, hospital stay before MV. Urinary antigens for Legionella and *Streptococcus pneumoniae*, and blood cultures were also performed.

The primary endpoints were: 1) the safety (need for urgent ETI) and effectiveness (changes in ABGs, Kelly-Matthay and cough scores) of FBO within two hours of NPPV; 2) the rate of major complications [24], especially septic complications and nosocomial pneumonia (included pulmonary aspiration) which were diagnosed using strict criteria [26,27]. Assuming a power of 80% with an α-error of 0.05, a sample size of 31 patients was calculated on the basis of the reported finding of major complications in NPPV compared with CMV-treated ARF patients with HE (30% vs. 65%) [10].

Secondary endpoints were: microbiological findings of BAL and relative adjustments of empiric antibiotic therapy, ABG changes, in-hospital mortality, tracheostomy, and length of hospital stay and MV.

### Statistical analysis

The Kolmogorov-Smirnov test was used to verify whether all recorded variables were normally distributed (*P *> 0.05). Continuous data are expressed as mean (standard deviation) if distributed normally or as median (interquartile range) if not; categorical data are presented as frequency. Continuous variables were compared with the two-tailed unpaired Student's *t *test (parametric data) or the Mann-Whitney *U *test (nonparametric data). Categorical data were compared using the chi-squared or, when appropriate, Fisher's exact test. A *P *value less than 0.05 was considered statistically significant. Analyses were performed using version 10.0 of SPSS software (SPSS, Chicago IL, USA).

## Results

During the study period, 38 and 33 eligible consecutive patients with acute decompensation of COPD triggered by CAP, showing depressed sensorium and bronchial hypersecretion, were admitted to the RSICU for NPPV and to the ICU for CMV, respectively. After careful matching (100% for all pre-defined parameters), 15 patients from each group were selected (Table 1); cases and controls did not differ significantly on any the variables used for matching. Concerning non-matching criteria, the two groups were similar except for age and Charlson score, which were significantly greater in the NPPV and CMV groups (Table 2).

**Table 1:** Matching criteria at baseline between the non-invasive positive pressure ventilation (NPPV) and the conventional mechanical ventilation (CMV) groups

	NPPV (n = 15)	CMV (n = 15)	*P*
pH, mean (SD)	7.27 (0.02)	7.27 (0.03)	0.858
PaO_2_/FiO_2_, mean (SD)	163 (60)	165 (13)	0.910
PaCO_2 _mmHg, mean (SD)	76 (7)	78 (13)	0.596
APACHE III score, mean (SD)	71 (9)	73 (6)	0.396
Kelly-Matthay score, mean (SD)	3.4 (1.2)	3.2 (1.0)	0.633
CAP, n involved lobes, median (IQR)	2 (1-2)	1 (1-2)	0.486
CURB-65 score, median (IQR)	3 (2-4)	3 (2-3)	0.806

APACHE, acute physiology and chronic health evaluation; CAP, community-acquired pneumonia; CURB-65, confusion, urea, respiratory rate, blood pressure, age 65 or over; FiO_2_, fraction of inspired oxygen; IQR, interquartile range; PaCO_2_, partial pressure of arterial carbon dioxide; PaO_2_, partial pressure of arterial oxygen; SD, standard deviation.

**Table 2:** Characteristics of the noninvasive positive pressure ventilation (NPPV) and the conventional mechanical ventilation (CMV) groups according to nonmatching criteria

	NPPV (n = 15)	CMV (n = 15)	*P*
Age, years, mean (SD)	80 (5)	73 (5)	0.001
Male, n (%)	12 (80)	9 (60)	0.427
BMI, Kg/m^2^, mean (SD)	25 (4)	26 (3)	0.509
FEV_1_, % of pred., mean (SD)^a^	30 (5)	31 (5)	0.866
FVC, % of pred., mean (SD)^a^	39 (8)	42 (8)	0.262
PaO_2_, mmHg, mean (SD)^a^	51 (3)	54 (3)	0.105
PaCO_2_, mmHg, mean (SD)^a^	59 (5)	57 (4)	0.257
pH, mean (SD)^a^	7.39 (0.02)	7.40 (0.03)	0.514
Charlson score, median (IQR)	2 (1-2)	1 (1-1)	0.041
Exacerbations, median (IQR)^b^	1.7 (0.9)	1.1 (1.0)	0.600
Antibiotic use, n (%)^c^	5 (33.3)	6 (40.0)	0.705
DNR order, n (%)	2 (13.3)	0 (0.0)	0.483
Pre-MV hospital, days median (IQR)	1 (1-2)	1 (1-2)	0.646
RR, bpm, mean (SD)	34 (4)	36 (3)	0.160
HR, bpm, mean (SD)	102 (12)	108 (12)	0.224
PS, cmH_2_O, mean (SD)	18 (4)	17 (4)	0.445

BMI, body mass index; DNR, do-not-resuscitate; FEV1, forced expiratory volume in the first second; FVC, forced vital capacity; HR, heart rate; IQR, interquartile range MV, mechanical ventilation; PaCO_2_, partial pressure of arterial carbon dioxide; PaO_2_, partial pressure of arterial oxygen; PS, pressure support; RR, respiratory rate; SD, standard deviation.

^a ^stable status within the previous 6 months.

^b ^within the previous 12 months.

^c ^within the previous 3 months.

The clinical physiological features of the 15 recruited cases did not differ from those of the 23 patients who were admitted to the RSICU and treated with NPPV but were not selected after the matching process (Table 3): this homogeneity excluded a bias due to lack of internal validity of the sample.

**Table 3:** Characteristics of patients receiving noninvasive positive pressure ventilation (NPPV) according if they were or were not recruited in the study

	Recruited (n = 15)	Non-recruited (n = 23)	*P*
Age, years, mean (SD)	80 (5)	79 (4)	0.803
Male, n (%)	12 (80)	14 (61)	0.294
BMI, Kg/m^2^, mean (SD)	25 (4)	25 (3)	0.537
Charlson score, median (IQR)	2 (1-2)	2 (1-1)	0,535
pH, mean (SD)	7.27 (0.02)	7.28 (0.01)	0.677
PaO_2_/FiO_2_, mean (SD)	163 (60)	169 (15)	0.988
PaCO_2 _mmHg, mean (SD)	76 (7)	75 (10)	0.647
APACHE III score, mean (SD)	71 (9)	70 (6)	0.804
Kelly-Matthay score, mean (SD)	3.4 (1.2)	3.2 (1.0)	0.633
CAP, n involved lobes, median (IQR)	2 (1-2)	1 (1-2)	0.883

APACHE, acute physiology and chronic health evaluation; BMI, body mass index;

CAP, community-acquired pneumonia; FiO_2_, fraction of inspired oxygen; IQR, interquartile range; PaCO_2_, partial pressure of arterial carbon dioxide; PaO_2_, partial pressure of arterial oxygen; SD, standard deviation.

**Figure 2:**
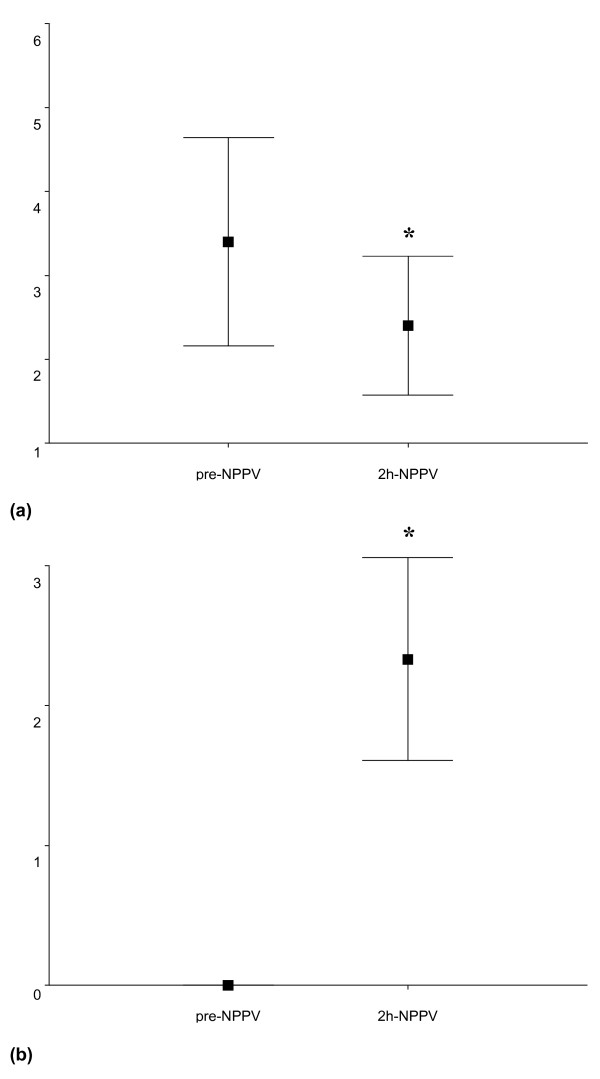
**(a) Changes of Kelly--Matthay and (b) cough efficiency score after two hours of noninvasive ventilation (NPPV)**. Values are expressed as mean (standard deviation). * *P *< 0.005 vs. baseline.

FBO was performed 18.5 (6.9) minutes after starting NPPV and lasted 7.8 (3.1) minutes with the removal of 23 (18) ml of respiratory secretions. No major complications, including the need for emergent ETI and pneumothorax, occurred before, during, or after FBO within two hours of NPPV. All patients tolerated FBO well while on NPPV. PaO_2_/FiO_2 _and SpO_2 _significantly increased before, during and after FBO compared with baseline, while the improvement in PaCO_2 _and pH became statistically significant after FBO in the NPPV group (Table 4). Both Kelly-Matthay and cough efficiency scores significantly improved after two hours of NPPV (Figure 2). Evaluation of sensorium and cough was not possible in the CMV group for the administration of sedation.

**Table 4:** Time course of arterial blood gases before, during and after fiberoptic bronchoscopy (FBO) in the noninvasive positive pressure ventilation (NPPV) group

	pre-NPPV	pre-FBO	during FBO	post-FBO^a^
pH, mean (SD)	7.27 (0.02)	7.29 (0.02)	7.29 (0.02)	7.37 (0.04)**
PaCO_2 _mmHg, mean (SD)	76 (7)	75 (8)	73 (7)	60 (3)**
PaO_2_/FiO_2_, mean (SD)	163 (60)	211 (39)**	203 (40)*	200 (70)**
SpO_2_, %, mean (SD)	79 (9)	93 (2)**	92 (3)**	92 (2)**
FiO_2_, mean (SD)	0.45 (10)	0.70	1.0	0.39 (12)

^a ^after 2 hours of NPPV.

* *P *< 0.05 vs pre-NPPV.

** *P *< 0.05 vs pre-NPPV.

FiO_2_, fraction of inspired oxygen; PaCO_2_, partial pressure of arterial carbon dioxide; PaO_2_, partial pressure of arterial oxygen; SD, standard deviation; SpO_2_, pulse oximetry.

BAL allowed the bacterial diagnosis of pneumonia in 80% of patients in the NPPV group and 60% of patients in the CMV group. The pattern of the isolated microorganisms was similar in the two groups. As a result of the BAL findings, the empiric antibiotic therapy was changed in 33.3% and 26.7% of NPPV and CMV-treated patients, respectively, (*P *> 0.05) due to de-escalation therapy (3 in the NPPV group and 2 in the CMV group) and multi-drug resistance of *S. pneumoniae *(1 in the NPPV group and 1 in the CMV group) and *P. aeruginosa *(1 in the NPPV group and 1 in the CMV group) (Table 5). Urinary pneumococcal antigen was positive in seven patients (4 in the NPPV group and 3 in the CMV group), while urinary antigen for Legionella was never identified. Blood cultures were only positive in three patients with pneumococcal pneumonia (1 in the NPPV group and 2 in the CMV group).

**Table 5:** Microbiological broncho-alveolar lavage (BAL) findings in the noninvasive (NPPV) and the conventional mechanical ventilation (CMV) groups

	NPPV (n = 15)^a^	CMV (n = 15)^b^	*P*
Diagnostic yield (>10^4 ^cfu/ml), n (%)	12 (80.0)	9 (60.0)	0.427
Change in antibiotic-therapy, n (%)	5 (33.3)	4 (26.7)	1.000
*Streptococcus pneumoniae, *n (%)	5 (33.3)	4 (26.7)	1.000
*Pseudomonas aeruginosa, *n (%)	4 (26.7)	3 (20.0)	1.000
*Staphylococcus aureus, *n (%)	2 (13.3)	1 (6.7)	1.000
*Haemophilus influenzae, *n (%)	2 (13.3)	2 (13.3)	1.000
*Enterobacteriaceae, *n (%)	1 (6.7)^c^	1 (6.7)^d^	1.000
*Moraxella catarrhalis, *n (%)	1 (6.7)	0 (0)	1.000

^a ^In three cases two microorganisms were isolated.

^b ^In two cases two microorganisms were isolated.

^c^*Escherichia coli*.

^d^*Klebsiella pneumoniae*.

**Figure 3:**
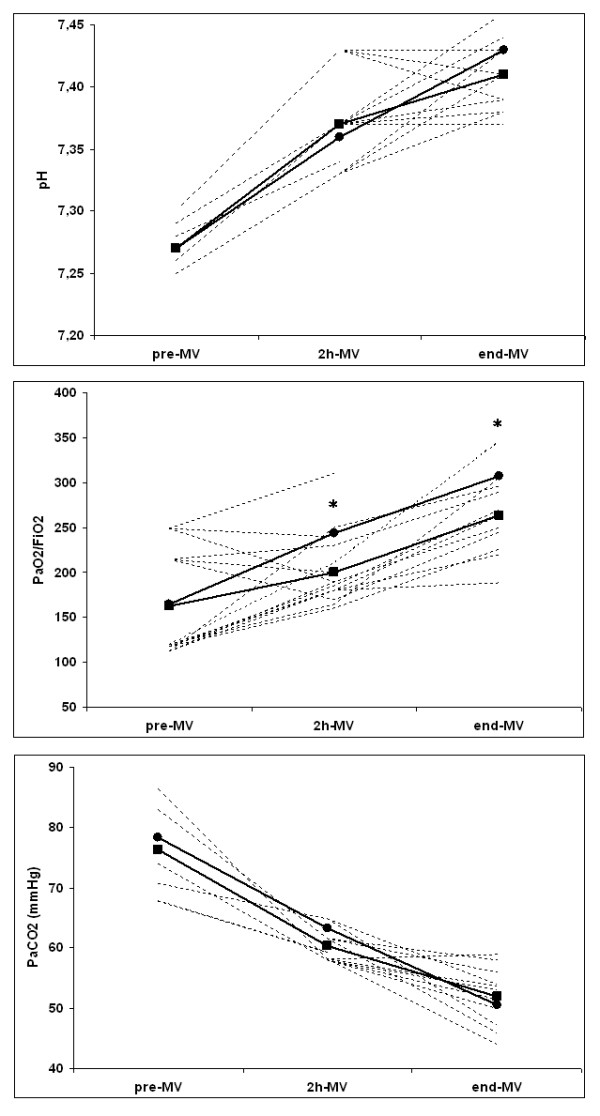
**Trend of mean values of arterial blood gases**. Values shown for before (pre-MV), after two hours (2 h-MV) and at the end of mechanical ventilation (end-MV) in the noninvasive positive pressure ventilation (NPPV) (Rectangles; pre-MV: n.15; 2 h-MV: n.15; end-MV: n.11) and the conventional mechanical ventilation (CMV) group (circles; pre-MV: n.15; 2 h-MV: n.15; end-MV: n.8). Dashed lines depicted the individuals values of pH, partial pressure of arterial oxygen (PaO_2_)/fraction of inspired oxygen (FiO_2_), partial pressure of arterial carbon dioxide (PaCO_2_) of NPPV-treated patients. * *P *< 0.05 between the groups.

Compared with baseline, pH and PaCO_2 _had improved similarly in the NPPV and CMV groups after two hours and at the end of MV. Conversely, PaO_2_/FiO_2 _level was significantly greater in the CMV group than in the NPPV group both after two hours and at the end of MV (Figure 3). Mean values of PS were similar after two hours (NPPV group: 18.2 (4.5) cmH_2_O; CMV group: 19.0 (5.6) cmH_2_O) and at the end of MV (NPPV group: 16.8 (7.9) cmH_2_O; CMV group: 17.3 (8.1) cmH_2_O).

The complication rate was significantly higher in the CMV than in the NPPV group due to a greater occurrence of infectious events. Pulmonary aspiration did not occur in the NPPV group. Tracheostomies were significantly fewer in the NPPV than in the CMV group. Hospital mortality, and length of hospitalisation and MV were similar in the two groups (Table 6).

**Table 6:** Hospital outcomes in the noninvasive (NPPV) and the conventional mechanical ventilation (CMV) groups

	NPPV (n = 15)	CMV (n = 15)	*P*
Tracheostomy, n (%)	0 (0)	6 (40.0)	0.008
Hospital mortality, n (%)	3 (20.0)	7 (46.7)	0.121
Complications, n (%)	3 (20.0)	12 (80.0)	0.001
Septic complications, n (%)	3 (20.0)	9 (60.0)	0.025
*Sepsis and septic shock, n (%)*	1 (6.7)	5 (33.3)	0.169
*Nosocomial pneumonia, n (%)*	2 (13.3)	4 (26.7)	0.651
Non-septic complications, n (%)	0 (0)	3 (20.0)	0.224
*Cardiovascular complications, n (%)*	0 (0)	1 (6.7)	1.000
*Acute renal failure, n (%)*	0 (0)	1 (6.7)	1.000
*Gastrointestinal bleeding, n (%)*	0 (0)	1 (6.7)	1.000
MV length, days median (IQR)	9 (5-12)^a^	17 (7-27)^b^	0.270
Length of hospitalisation, days mean (SD)	20 (4)^a^	27 (21)^b^	0.423

IQR, interquartile range; MV, mechanical ventilation; SD, standard deviation.

^a^NPPV patients who avoided endotracheal intubation (n = 12).

^b ^CMV patients who survived to discharge (n = 8).

NPPV failed in 3 of 15 patients (20%) after 17.3 (9.5) days and a total of 216.0 (109.3) hours of ventilation due to worsening ABGs (n = 2) and secretion retention (n = 1). All of them developed septic complications after ETI and died due to septic shock (n = 2) or cardiac arrest (n = 1). Mild facial skin erythema occurred in four patients.

Causes of in-hospital death in the CMV group were septic shock (n = 5), acute renal failure (n = 1), cardiac arrest (n = 1), and after tracheostomy (n = 3).

## Discussion

This is the first study to verify the safety and effectiveness of the early therapeutic FBO during NPPV. It also compared the outcomes of this strategy with ETI and CMV in COPD decompensations for CAP in patients who were not considered appropriate candidates for NPPV because of HE and an inability to spontaneously eliminate an excessive burden of secretions. Two hours of NPPV associated with FBO-based mucous clearance significantly improved ABGs, sensorium and cough efficiency without any complications. Improvement in PaCO_2 _and pH, as well as hospital mortality, and durations of hospitalisation and MV were similar in the NPPV and CMV groups. Interestingly, NPPV significantly reduced serious infectious complications compared with CMV.

The main limitation of the present study is the case-control design and lack of randomisation, which may bias results in favour of the treatment under investigation. Both cases and controls were prospectively enrolled during the same period, but unfortunately we could not perform a RCT because CMV could be applied only in the ICU, and the unpredictability of bed availability in different units made randomisation difficult. Furthermore, even if available in both units, NPPV was mainly applied in the RSICU to treat exacerbations of chronic respiratory disorders for cost-utility advantages [28]; conversely, in ICU there was a large priority to admit complex and severe *de novo *acute diseases requiring both invasive monitoring and CMV (i.e. post-surgery ARF, multi-organ failures, poly-traumas etc.). On the other hand, well-designed observational studies may yield reliable results when cases and controls are well balanced by careful matching, and the interference of confounding factors is minimised [29]. In our study, cases were similar to controls not only for the matching criteria (ABGs, APACHE III score, sensorium and severity of CAP) but also for other historical clinical physiological features. Conversely, the older age and more numerous comorbidities of the NPPV group could have favoured the CMV treatment. Moreover, the clinical physiological homogeneity between the cases and NPPV patients who were not recruited after the matching process excluded a bias of selection.

Another concern regarding the study design is the different setting where the two groups were treated. However, the lower intensity of care in the RSICU compared with the ICU (e.g., nurse:patient ratio) could have contributed to a higher rate of NPPV failures. Moreover, the application of the same inclusion and exclusion criteria as well as the same standard therapies and nursing activities (i.e. cough and sensorium assessment) in the two units belonging to the same hospital should have minimised this bias. Another point is the implementation of shared intra-hospital multi-disciplinary protocols for the management of severe COPD exacerbations. It should also be emphasised that our RSICU has acquired a high level of expertise with NPPV and FBO techniques, including for patients with HE [9,10], with the capability of promptly intubating failing patients. Thus our findings may not be reproducible in units less experienced in NPPV.

Finally, a criticism may be also directed to the choice of CMV-treated patients as a control group. It could be argued that a comparison between NPPV plus FBO vs NPPV alone may have been a more appropriate design to address the clinical outcomes of the study. This would be true for COPD patients in an earlier phase of their exacerbation compared with those of our study who were not clearly eligible for NPPV for their severe sensorium and cough depression [1,2]. The risk connected with a delayed intubation would not have been justified in selected COPD patients with a high chance of NPPV failure. However, a trial comparing NPPV combined or not with FBO in less severe COPD decompensations with impaired mucous clearance could be of help to integrate the findings to the present study.

Due to the lack of direct access to the airways, NPPV is not appropriate when patients are incapable of spontaneously removing abundant secretions. A rate of NPPV failure of 61% was reported in 23 patients with ARF of different aetiology showing copious secretions [4]. Moreover, in two series of COPD exacerbations with moderate-to-severe HE [9,10], the inefficient clearance of secretions caused 33% and 43% of all NPPV failures, respectively. Interestingly, Conti and colleagues [24] showed that in two of the nine COPD patients who required ETI within two to six hours of ventilation, NPPV failed for the difficult management of copious secretions. As a matter of fact, the possibility of clearing the airways in the early phases of NPPV is likely to reduce the need for ETI in patients with an unfavourable balance between an excessive burden of secretions (e.g. COPD exacerbations due to CAP) and an inefficient spontaneous clearance (e.g. poor cough reflex due to HE).

The most innovative finding of our study is the successful application for the first time of early FBO as a mucous clearance technique under NPPV in patients with COPD exacerbations who should have been intubated for their inability to cope with copious secretions and their altered level of consciousness [1,2]. This is particularly true for CAP-triggered COPD decompensations, which represent a 'challenging' model in terms of the burden of secretions also involving the distal airways. With this strategy, FBO-based suction of accumulated secretions during ventilation facilitated NPPV to improve ABGs, sensorium level and cough efficiency. The quick favourable effect of such a therapeutic approach is not surprising because it acts by interrupting the vicious circle triggered by the coexistence of abundant secretions and sensorium depression. However, the application of the common therapist manoeuvres to manage secretions in critical respiratory patients was essential for the success of the FBO-NPPV strategy in our RSICU [30]. This may explain why we did not need to repeat the FBO procedure for the recurrence of mucous accumulation in all but one case who failed NPPV after 10 days of ventilation.

The lack of major complications is consistent with previous reports, which clearly demonstrated the feasibility and safety of FBO plus BAL performed under the assistance of NPPV in patients with severe hypoxaemic and hypercapnic ARF who should have to be otherwise intubated to allow such invasive procedures [12-17]. Therefore, it should be underlined, as the novelty of our study, the safe use of FBO in patients with severe ARF requiring a mandatory ventilatory support because no relevant complications (cardiovascular, pneumothorax, emergent intubation) were reported. In contrast, in previous studies [12-17] NPPV was applied with the aim to prevent ABGs deterioration and the need for ETI during the BAL performed in spontaneously breathing patients.

Although few articles have reported the efficacy of non-invasive techniques in augmenting sputum production in exacerbated alert COPD patients [5-7], these options may not have been successful in patients with severe COPD exacerbations with depressed sensorium and inefficient cough.

In agreement with previous experiences, our study confirms the high success rate of NPPV when applied in patients with hypercapnic ARF and reduced levels of consciousness [8-10]. The finding of a relatively low rate of ETI in our study parallels the results of a previous RCT, which highlighted the different outcomes of NPPV in CAP as a cause of ARF, depending on if ventilation was delivered to COPD or non-COPD patients [11].

The quick improvement of the sensorium level and of the cough reflex together with the avoidance of ETI and the less aggressive monitoring associated with NPPV management in RSICU may have contributed to the lower occurrence of septic nosocomial complications in the NPPV group than in the CMV group. This is not unexpected as the effectiveness of NPPV in preventing ETI-correlated complications (i.e. nosocomial pneumonia and VAP), especially in patients with chronic respiratory disorders, has been clearly demonstrated [31]. Furthermore, it could be speculated that the different occurrence of nosocomial infections in the two groups may at least in part justify the finding of fewer trachestomies in the NPPV vs the CMV group; this hypothesis seems to be suggested by the trend towards a longer duration of MV in patients who developed septic complications compared with these who did not (20 (6 to 28) vs 7 (5 to 12) days; *P *= 0.267). However, this data should be interpreted with caution as the study was not powered for assessing the differences in the rate of tracheostomy between the groups.

The high diagnostic yield of BAL for the identification of the bacterial aetiology of CAP both in NPPV (80%) and in CMV-treated patients (60%) is difficult to compare with previously published data (36 to 78%) [32-34] due to the large heterogeneity existing among the different studies in terms of severity of CAP, underlying diseases, need for MV, previous use of antibiotics, and hospital setting. We decided to perform BAL to have more chances to achieve a microbial diagnosis as an adequate sputum sample was not easily available in such critical patients. The spectrum of microorganisms isolated in our study belongs to the typical bacterial pattern of CAP found in patients with COPD at advanced stages [35,36]. Concerning the change of the initial empirical antibiotic therapy on the basis of BAL results, the rate of 26.7% and 33.3% found in the CMV and NPPV groups, respectively, is consistent with a recent finding of 27.3% reported in immunocompetent CAP patients [32]. However, the clinical meaning of this result should be scaled down if we consider that the changes of the initial therapy were not due to the inadequacy of the empiric approach recommended by the guidelines for such COPD patients with severe CAP [19] but to the de-escalation strategy and occurrence of drug resistance.

Some caveats are worth considering when interpreting our results. Patients with HE and mucous accumulation due to severe COPD exacerbations are critically ill and warrant close observation in a skilled unit with the means to promptly intubate the patient if necessary. Thus the application of NPPV to treat severe ARF with the concomitant use of FBO to remove abundant secretions should be reserved for centers where all staff members have acquired sufficient experience with these kinds of patients and these kinds of procedures [37].

## Conclusions

In this pilot study we have shown that in acute COPD decompensations due to CAP in patients who are not considered appropriately eligible for a noninvasive ventilatory approach because of HE and an inability to spontaneously clear copious secretions, NPPV with early FBO performed by an experienced team is feasible, safe and effective. Importantly, this innovative strategy was not associated with relevant complications, such as emergent intubation, cardiovascular events and pneumothorax. Moreover, compared with CMV, this new approach reduces nosocomial infections associated with ETI. Even if this NPPV strategy may be a successful alternative to CMV to manage selected COPD patients within expert units with prompt access to ETI, larger RCTs are necessary to confirm this preliminary result and, therefore, to test the efficacy of the FBO-NPPV protocol in a single center applied to an earlier time-course of COPD decompensations when ETI is not mandatory.

## Key messages

• NPPV with early FBO performed by an experienced team is a potential alternative to ETI in acute COPD decompensations with HE and inability to clear copious secretions.

## Abbreviations

ABGs: arterial blood gases; APACHE: acute physiology and chronic health evaluation; ARF: acute respiratory failure; ATS: American Thoracic Society; BAL: broncho-alveolar lavage; CAP: community-acquired pneumonia; cfu: colony forming units; CMV: conventional mechanical ventilation; COPD: chronic obstructive pulmonary disorder; DNR: do-not resuscitate; ETI: endotracheal intubation; FBO: fiberoptic bronchoscopy; FiO2: fraction of inspired oxygen; HE: hypercapnic encephalopathy; IDSA: Infective Diseases Society of America; MV: mechanical ventilation; NPPV: non-invasive positive pressure ventilation; PaCO_2_: partial pressure of arterial carbon dioxide; PaO_2_: partial pressure of arterial oxygen; PEEP: positive end-expiratory pressure; PS: pressure support; RCTs: randomised controls trials; RR: respiratory rate; RSICU: semi-intensive respiratory care unit; SpO_2_: pulse oximetry.

## Competing interests

The authors declare that they have no competing interests.

## Authors' contributions

RS had the idea for the study and helped with its design, contributed to data collection, analysed and interpreted data, and wrote the report. MN and UM contributed to data collection and to revise the manuscript.
